# Quantitative User Data From a Chatbot Developed for Women With Gestational Diabetes Mellitus: Observational Study

**DOI:** 10.2196/28091

**Published:** 2022-04-18

**Authors:** Mari Haaland Sagstad, Nils-Halvdan Morken, Agnethe Lund, Linn Jannike Dingsør, Anne Britt Vika Nilsen, Linn Marie Sorbye

**Affiliations:** 1 Department of Health and Caring Sciences Faculty of Health and Social Sciences Western Norway University of Applied Sciences Bergen Norway; 2 Department of Obstetrics and Gynecology Haukeland University Hospital Bergen Norway; 3 Department of Clinical Science University of Bergen Bergen Norway; 4 Norwegian Research Centre for Womens´s Health, Rikshospitalet Oslo University Hospital Oslo Norway

**Keywords:** chatbot, gestational diabetes mellitus, user data, log review, eHealth, diabetes, pregnancy, dialogue

## Abstract

**Background:**

The rising prevalence of gestational diabetes mellitus (GDM) calls for the use of innovative methods to inform and empower these pregnant women. An information chatbot, Dina, was developed for women with GDM and is Norway’s first health chatbot, integrated into the national digital health platform.

**Objective:**

The aim of this study is to investigate what kind of information users seek in a health chatbot providing support on GDM. Furthermore, we sought to explore when and how the chatbot is used by time of day and the number of questions in each dialogue and to categorize the questions the chatbot was unable to answer (fallback). The overall goal is to explore quantitative user data in the chatbot’s log, thereby contributing to further development of the chatbot.

**Methods:**

An observational study was designed. We used quantitative anonymous data (dialogues) from the chatbot’s log and platform during an 8-week period in 2018 and a 12-week period in 2019 and 2020. Dialogues between the user and the chatbot were the unit of analysis. Questions from the users were categorized by theme. The time of day the dialogue occurred and the number of questions in each dialogue were registered, and questions resulting in a fallback message were identified. Results are presented using descriptive statistics.

**Results:**

We identified 610 dialogues with a total of 2838 questions during the 20 weeks of data collection. Questions regarding blood glucose, GDM, diet, and physical activity represented 58.81% (1669/2838) of all questions. In total, 58.0% (354/610) of dialogues occurred during daytime (8 AM to 3:59 PM), Monday through Friday. Most dialogues were short, containing 1-3 questions (340/610, 55.7%), and there was a decrease in dialogues containing 4-6 questions in the second period (*P*=.013). The chatbot was able to answer 88.51% (2512/2838) of all posed questions. The mean number of dialogues per week was 36 in the first period and 26.83 in the second period.

**Conclusions:**

Frequently asked questions seem to mirror the cornerstones of GDM treatment and may indicate that the chatbot is used to quickly access information already provided for them by the health care service but providing a low-threshold way to access that information. Our results underline the need to actively promote and integrate the chatbot into antenatal care as well as the importance of continuous content improvement in order to provide relevant information.

## Introduction

Gestational diabetes mellitus (GDM) is defined as glucose intolerance that arises and is discovered during the second or third trimester of pregnancy [1]. Globally, it affects 1 in 7 pregnant women [2]. In 2019, 5.09% (2769 of 54,407) of pregnant women were diagnosed with GDM in Norway [3], but the condition is assumed to occur in up to 10% of all Norwegian pregnancies, varying by ethnic origin [4,5]. GDM is associated with numerous pregnancy complications affecting both the mother and the fetus [2,6-8], and women with GDM have an increased risk of later developing type 2 diabetes [9,10]. To ensure good health for both the mother and the fetus, a thorough follow-up of women with GDM is required. In reducing the consequences of GDM [7,8,11-13], antenatal training in self-managing blood glucose measurements and nutritional and physical activity education are cornerstones in current clinical care [8,14,15]. Follow-up is provided both by the primary and specialist health care based on blood glucose values [15]. Information provided should aim to strengthen the women’s autonomy to cope with the diagnosis, enabling them to make the best decisions for their own health [8,16]. Traditionally, information is provided in person by medical professionals in addition to written information and referral to official websites. Studies indicate that women with GDM experience a lack of personally adapted information, which may contribute to a sense of insecurity [17,18]. This calls for new ways of complementing the established care. Furthermore, the rising prevalence of GDM will likely increase the need for antenatal care consultations [15], and the use of new technologies like chatbots could be a valuable asset in future health care [19].

Use of information and communications technology has the potential to improve public health by increasing efficiency, lowering costs, and improving quality of care [20]. Different health technology solutions may have positive effects on the self-management of diabetes [19], an asset especially important for women with GDM. Chatbots are conversational agents based on artificial intelligence that interact with users in a natural language, either text-based or voice driven, independent of time and location [21,22]. There is a growing number of health chatbots developed for different purposes [23]. Developed by health care personnel and users, Dina, Norway’s first health chatbot, was launched in 2018 and made freely available for women with GDM [24]. Dina provides information on GDM and relevant topics related to the condition in accordance with national recommendations. Chatbots providing health information and support regarding other specific conditions have been developed elsewhere [25-27].

Health technology solutions such as chatbots are rarely implemented in health care after the initial pilot study phase [19]. Previous studies evaluating health chatbots have mostly used interviews or questionnaires and have not been based on quantitative analysis of chatbot logs [28]. Exploring chatbot dialogues may provide valuable information needed for further improvement [29]. In 2019, Dina the chatbot was integrated into the Norwegian official digital health platform and is thus an example of implementation of health technology solutions in clinical care.

Our study aim is to provide a basis for improvement and further development of Dina the chatbot by exploring log data on what type of information the users’ seek, with the research question being as follows: What type of information do users seek in a health chatbot providing support on GDM?* *Further specific aims are to explore how many questions each dialogue contains and the time of day the chatbot is used. We subcategorized questions that led to a fallback message from the chatbot to obtain a deeper understanding of which type of questions the chatbot was unable to answer. This knowledge may provide insight into the use of health chatbots and potentially establish more general theoretical knowledge on this type of chatbot.

## Methods

### Background and Settings for Developing the Chatbot

In 2016, a pilot study for the project revealed an incoherent follow-up and lack of personally adapted information provided to women with GDM. Contrary to current practice when promoting health technology solutions [30], Dina the chatbot was developed after an observed and expressed need from both women with GDM and involved clinicians. Dina the chatbot was developed at Haukeland University Hospital in cooperation with Bergen municipality and Western Norway University of Applied Sciences. User involvement throughout development and evaluation of health technology is important [19], and a user-centered design [31] was applied throughout the development process. User representatives were involved from idea conception to evaluation [32,33] as was an interdisciplinary team of gynecologists, midwives, psychologists, nutritionists, endocrinologists, and information technology developers [34]. Initially, the chatbot was launched at its own website and was made available to all pregnant women in the country, but promotion was limited to Haukeland University Hospital and surrounding municipalities. In 2019, Dina the chatbot was implemented in the official Norwegian digital health platform, presented with an improved user interface (Dina 2.0), and hence made more available to all pregnant women in Norway. Of the 54,407 women who gave birth in Norway in 2019, the target population represented 2769 pregnant women diagnosed with GDM although a portion of these women might have had difficulty using the chatbot due to language barriers. The chatbot offers low-threshold access to quality-checked information, as login or a registered user account is not required. The overall goal for developing the chatbot was to provide reliable information to women with GDM, strengthen women’s knowledge about their own health, and improve their daily coping with the condition. Dina is intended as an addition to established care and was developed as an informational chatbot. Pregnant women frequently seek information online and from apps [35] and expect modern health service to provide integrated digital solutions in treatment and follow-up [36]. This chatbot could be an important supplement for pregnant women with GDM [35]. However, evaluation of its use based on objective data from the chatbot’s log is needed for further development. The results may also be beneficial in future development of similar informational chatbots created for other specific medical conditions. An observational study analyzing user data from Dina the chatbot was designed. Dialogues were collected from the chatbot’s log and platform over 20 weeks (from week 41 to 48 in 2018 and from week 47 in 2019 to week 6 in 2020). The management team of Dina added “test dialogues” to the chatbot for training and further development. However, these test dialogues were excluded from the collected data because they were not raised by the target population of Dina the chatbot and would have biased the results. A manual log review of the collected dialogues was performed. All data were anonymous, and the identification of users was not permitted. Thus, it was impossible to identify unique users or to determine if they visited the chatbot once or several times. Each dialogue served as the unit of analysis, and we prefer using the term “users of the chatbot” rather than “women with GDM.”

### Variables

**Figure 1:**
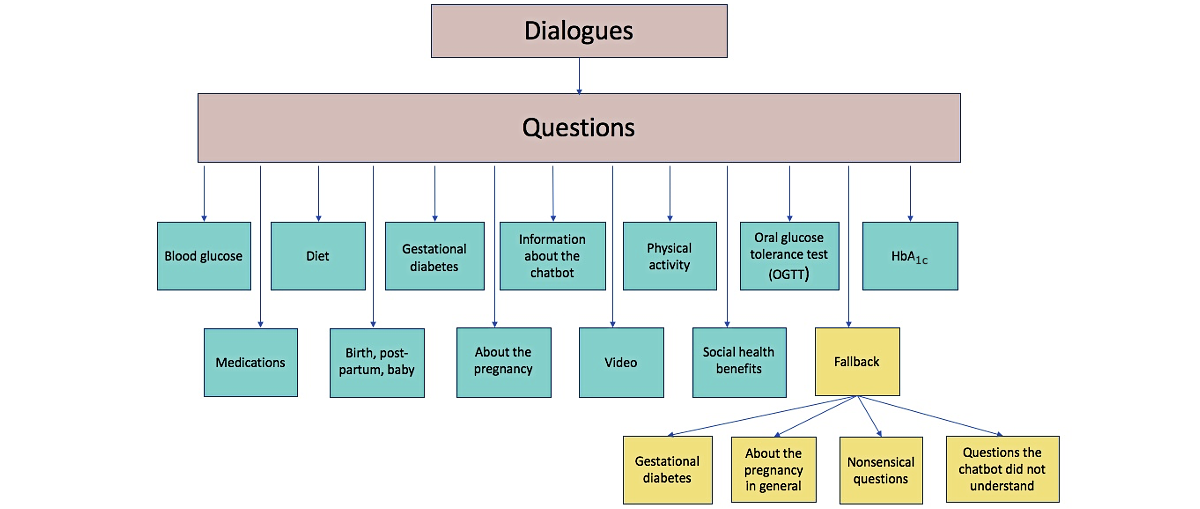
Categorization of questions for Dina the chatbot. HbA_1c_: hemoglobin A_1c_.

Questions from users to the chatbot were categorized (see Figure 1), and all categories were mutually exclusive [37].

We identified the time of day that dialogues took place and the number of questions in each dialogue.

Table 1 presents the variables in Dina the chatbot with explanations and categories.

The time of day the dialogue took place was grouped into day (8 AM-3:59 PM), afternoon (4 PM-11:59 PM), and night (midnight-7:59AM), while the number of questions in each dialogue was categorized into 1-3, 4-6, 7-9, and ≥10 questions. We registered the frequency of questions the chatbot was unable to answer that resulted in a fallback message from the chatbot (eg, “I´m sorry, I cannot answer that at this point, could you rephrase the question?”). Questions resulting in a fallback message were subcategorized and counted numerically (see Figure 1) to explore which categories of questions the chatbot was unable to answer.

**Table 1:** Variables with explanations for Dina the chatbot.

Variable	Explanation	Categorization
Dialogue (the unit of analysis)	Question sequence between the user and the chatbot	N/A^a^
Period	Data collection period	Period 1: week 41 to 48 in 2018 Period 2: week 47 in 2019 to week 6 in 2020
Week number	Week the dialogue took place	Weeks 41 to 48 in 2018 Week 47 in 2019 to week 6 in 2020
Day of the week	Day of the week the dialogue took place	Monday, Tuesday, Wednesday, Thursday, Friday, Saturday, Sunday
Date	Date the dialogue took place	Day, month, year
Time of day	Time of day the dialogue took place	8 AM-3:59 PM 4 PM-11:59 PM Midnight-7:59AM
Total number of questions in each dialogue	Number of questions from the user to the chatbot in each dialogue	1-3, 4-6, 7-9, ≥10
Blood glucose	Number of questions from the user regarding blood glucose	Numerical
Diet	Number of questions from the user regarding diet and nutrition	Numerical
GDM^b^	Number of questions from the user regarding the GDM diagnosis	Numerical
Information about the chatbot	Number of maneuvers to “navigate” in the chatbot (theme button, general information about the chatbot, privacy in use, greetings)	Numerical
Physical activity	Number of questions from the user regarding physical activity	Numerical
HbA_1c_^c^ (average blood glucose levels)	Number of questions from the user regarding HbA_1c_ (specific test used to diagnose pre-existing diabetes before the 16th week of pregnancy)	Numerical
OGTT^d^	Number of questions from the user regarding OGTT	Numerical
Medications	Number of questions from the user regarding medications used in the treatment of GDM	Numerical
Birth/postpartum/baby	Number of questions from the user regarding birth, postpartum period, and/or the baby	Numerical
About the pregnancy	Number of questions from the user regarding the pregnancy in general	Numerical
Video	Link to informational videos	Numerical
Social health benefits	Number of questions from the user regarding sick leave and appointments with midwife or doctor	Numerical
Fallback	Number of questions in free text from the user the chatbot failed to answer that resulted in a fallback message from the chatbot (eg, “I’m sorry, I cannot answer that at the time, please contact your physician or midwife for further information.”)	Numerical Questions about GDM Questions about the pregnancy in general Questions the chatbot did not understand (eg, questions with several spelling errors, questions asked in a foreign language) Nonsensical questions (eg, “Who is the king of Denmark?”, “When does my bus leave?”)

^a^N/A: not applicable.

^b^GDM: gestational diabetes mellitus.

^c^HbA_1c_: hemoglobin A_1c_.

^d^OGTT: oral glucose tolerance test.

### Data Analyses

The unit of analysis was the individual dialogue. Dialogues were manually registered and thoroughly read by the first author (MHS). In dialogues, users could either type their questions in free text or click theme buttons. Dialogues could therefore consist of free-text questions, theme button questions, or a mix of the 2. All dialogues are displayed the same way in the chatbots log, making it impossible to distinguish between the predefined questions and free-text questions. Therefore, it was not possible to determine if a user chose to spell “blood sugar” in free text or if they pressed the theme button “blood sugar.” Both free-text questions and predefined questions were counted as a whole, but questions that led to fallback would naturally be free-text questions, as the chatbot offers answers to the questions that are predefined. Differentiation between free-text and theme button questions will be considered in the future development of the chatbot. Variables were coded as nominal or interval. We used descriptive statistics: frequencies, proportions, and percentages. For continuous variables, we have reported mean as the central tendency with SD. Normal distribution was tested by Q-Q plot. Chi-square tests were performed to explore associations between variables, and independent *t* tests were used for comparisons of means. Results are presented visually in charts [37]. IBM SPSS Statistics (version 26) was used for all analyses. The significance level was set at 5%.

### Ethical Considerations

This study was conducted as a collaboration between Haukeland University Hospital, Western Norway University of Applied Sciences, and Bergen municipality. The chatbot’s platform and technological design have been validated in a risk and vulnerability analysis by the technical department at Haukeland University Hospital, and users’ data are protected according to the General Data Protection Regulation [38]. Data are stored at a secure server, and access to data are limited to authorized personnel only. Users were informed by the chatbot about anonymity, asked not to leave any personal information, and told about potential future use of data for scientific purposes. This study was presented to the regional ethics committee of Western Norway (approval #167012, 12.08.2020) and found exempt from extended application, as all data are completely anonymous. The data protection officer of Haukeland University Hospital approved the study on August 25, 2020 (ID 1555).

## Results

A total of 610 dialogues containing 2838 questions were registered during data collection. In the first period, 288 dialogues were registered, containing 1329 questions, while in the in the second period, 322 dialogues were registered, containing 1509 questions.

### The Users’ Informational Needs

**Figure 2:**
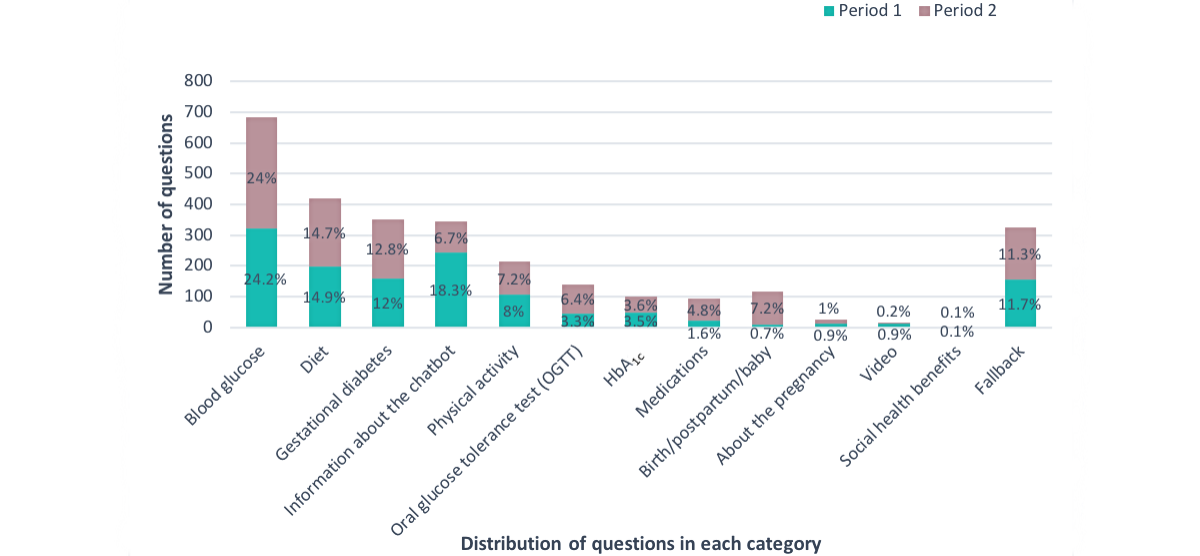
Number of questions by category and period (period 1: weeks 41 to 48 in 2018 with 1329 questions; period 2: week 47 in 2019 to week 6 in 2020 with 1509 questions) for Dina the chatbot.

Questions by category and period are presented in Figure 2.

Questions on blood glucose, diet, the GDM diagnosis, and physical activity accounted for 58.81% (1669/2838) of all questions, with little variation by period. The most frequent single category was questions on blood glucose levels, accounting for 24.07% (683/2838) of all questions. Questions on maneuvering and orienting in the chatbot (information about the chatbot) decreased from 18.28% (243/1329) in the first period, to 6.69% (101/1509) in the second period (*P*<.001). The remaining categories involving screening for GDM (oral glucose tolerance test and hemoglobin A_1c_), birth and postpartum period, treatment (medications), general information concerning the pregnancy, informational videos, and questions on social health benefits represented 17.58% (499/2838) of questions.

### When and How the Chatbot Was Used

**Figure 3:**
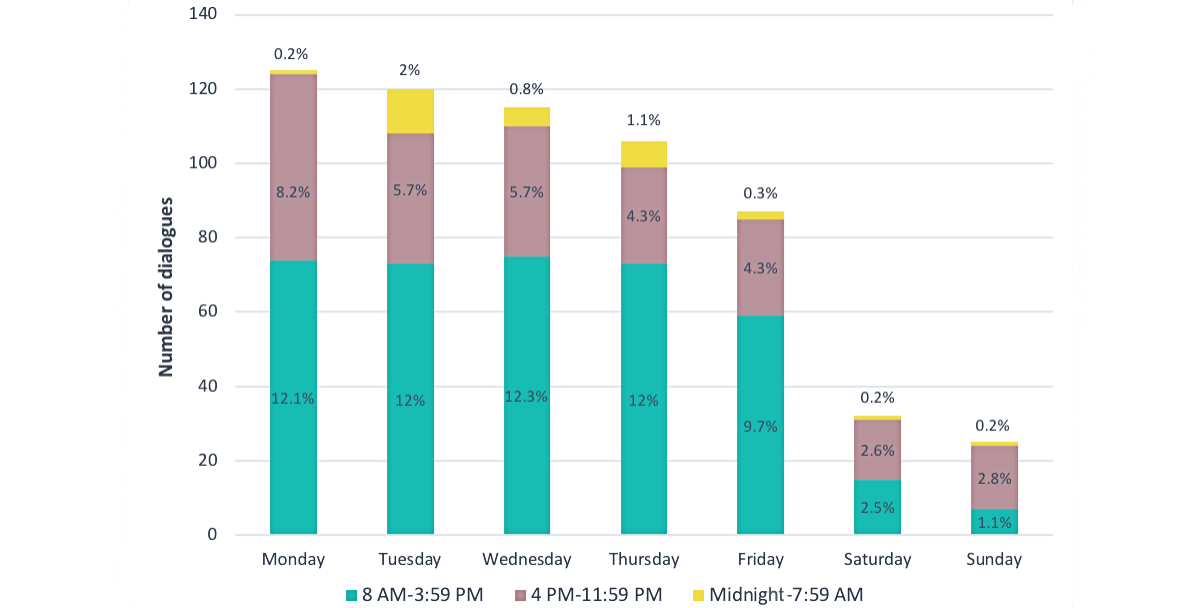
Number and percentage of dialogues in Dina the chatbot (n=610) by weekday and time of day for period 1 and period 2 combined.

The number of dialogues per week ranged from 5 to 92 across the 20 weeks of registration, with a mean value of 36 (SD 19.26) and 26.8 (SD 24.34) for the first and second period, respectively. The dialogues by day of the week and time of day for the 2 periods combined are presented in Figure 3.

In total, 90.7% (553/610) of all dialogues took place Monday through Friday, and 58% (354/610) took place during the daytime (8 AM-3:59 PM). The dialogues registered during the afternoon accounted for 28.2% (172/610) of dialogues Monday through Friday. There was little registered activity during weekends.

**Figure 4:**
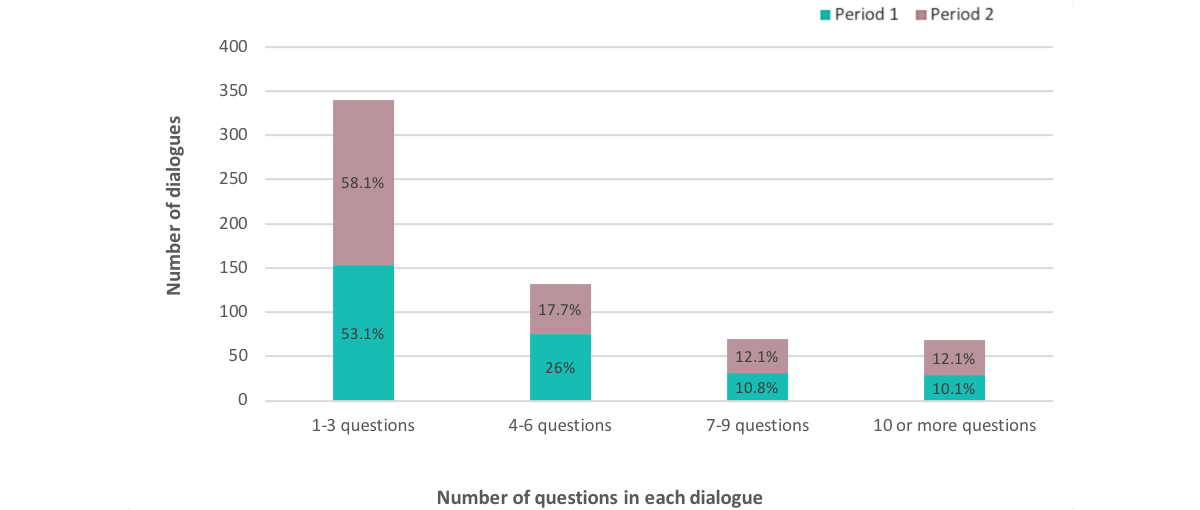
Dialogues by number of questions and period (period 1: weeks 41 to 48 in 2018, 288 dialogues; period 2: week 47 in 2019 to week 6 in 2020, 322 dialogues) for Dina the chatbot.

The number of questions in each dialogue ranged from 1 to 38, with a mean value of 4.65 for the 20 weeks of registration. Short dialogues (1-3 questions) were most frequent, both in the first (153/288, 53.1%) and second period (187/322, 58.1%; see Figure 4).

There was a decrease in number of dialogues containing 4-6 questions from the first period (75/288, 26.0%) to the second period (57/322, 17.7%; *P*=.013). Long dialogues of >7 questions were stable across the 2 periods.

### Ability To Answer Questions and Fallback by the Chatbot

**Figure 5:**
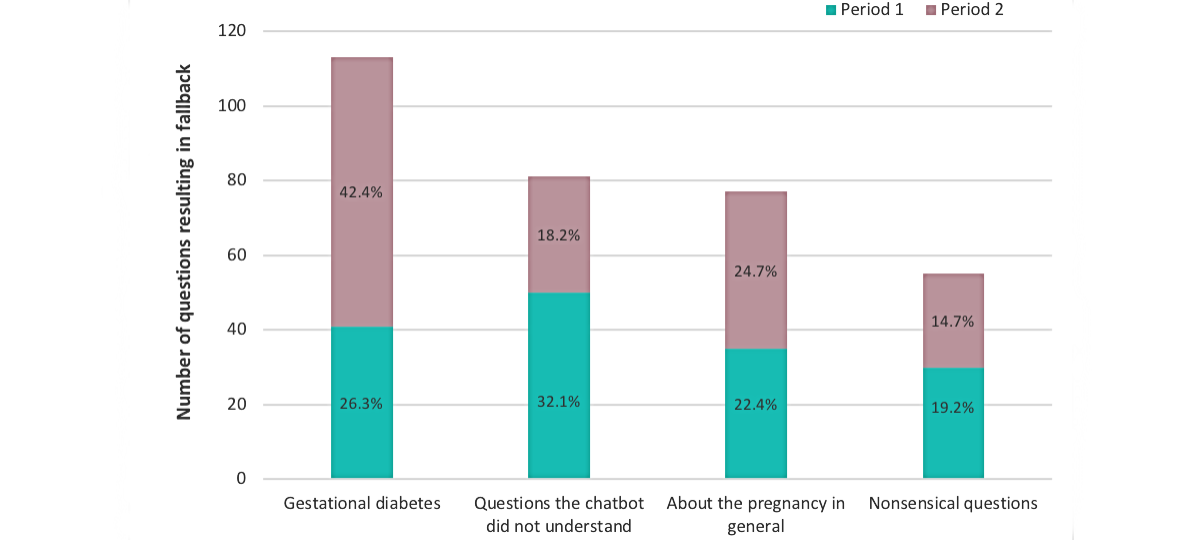
Types of fallback question by period (period 1: weeks 41 to 48 in 2018, n=156; period 2: week 47 in 2019 to week 6 in 2020, n=170) for Dina the chatbot.

Overall, Dina the chatbot was able to answer 88.15% (2512/2838) of all questions asked by users. Figure 5 shows the types of fallback questions by period.

Fallback questions on GDM increased from 26.3% (41/156) to 42.4% (72/170) from the first to the second period (*P*=.002), while the fallback questions the chatbot did not understand decreased from 32.1% (50/156) to 18.2% (31/170; *P*=.004). Fallback questions about pregnancy in general and nonsensical questions showed small variations between the 2 periods.

## Discussion

### Principal Findings

Nearly 60% (1669/2838) of all questions from users were on blood glucose, diet, GDM, and physical activity, and the chatbot was able to answer 88.51% (2512/2838) of all posed questions. The chatbot was most frequently used during the daytime, Monday through Friday, and most dialogues were short, containing 1-3 questions. However, the mean number of dialogues per week was 36 in the first period and 26.83 in the second period.

#### Most Frequently Asked Questions to Dina the Chatbot

Few prior studies have evaluated health chatbots based on log reviews [28]. Inkster et al [27] explored log data on self-reported symptoms of depression, using questionnaires integrated in their chatbot, Wysa, but with a different focus than ours. To the extent of our knowledge, our study is the first study aiming to explore what users ask a health chatbot integrated into a national health service platform by categorizing incoming questions. Dina the chatbot provides the opportunity to click on related themes and questions, “guiding” the user through the conversation. This might have increased conversation efficiency and influenced findings in this study by making some information more available for the users than other topics. Our results indicate that users mainly seek information with high relevance for their currently experienced issues related to GDM (questions regarding blood glucose levels and diet) as opposed to relevant information on future events like the postpartum period or contextual factors such as social health benefits. The information sought also seems to overlap with information available through the established antenatal health care program in Norway, as distribution of the most frequent questions mirrors the cornerstones in the treatment of GDM [14,15]. This indicates that the chatbot could serve as a low-threshold addition to the already-established health care service. The chatbot may enhance the treatment of GDM, promote stable blood glucose, and thereby prevent the development of adverse outcomes for the mother and the fetus. Qualitative studies have shown that women diagnosed with GDM may perceive a lack of personally adapted information, contributing to a feeling of insecurity [17,33] in which managing blood glucose measurements and changing diets are the main challenges [18]. Information provided from a chatbot can thus serve as a reminder or a confirmation for the user on already-received information from medical professionals [33] and not as a substitute for the traditional face-to-face consultation [39]. For users, adding technology like informational chatbots to the standard patient care may reduce insecurities [33,40] and potentially contribute to increased self-efficacy [19].

#### What Can User Behavior in the Chatbot Tell Us?

Despite some unanswered questions, we found that exploring user behavior in the chatbot will provide useful information for planning and organizing future antenatal care. A previous study on a comparable supportive chatbot developed for patients with breast cancer explored user behavior by asking a weekly question and by observing the retention rate among users [25]. As all data in our study were anonymous, we were unable to explore retention rate, and we treated each dialogue as the unit of analysis. We anticipated a higher frequency of use out of office hours, when medical professionals are less available. Surprisingly, the chatbot was most frequently used during the daytime, Monday through Friday. Even though a great advantage of chatbots is their 24-7 availability, frequent daytime use provides valuable insight for planning future antenatal care. To our knowledge, prior studies on health chatbots have not explored this issue before. The frequent use during the daytime may be a result of the users needing to quickly access information already provided for them by the health care service. With the timeframe of consultations often being limited [41], questions may arise before or after consultations [33]. The chatbot may provide reassurance for managing the condition of variable validity as an alternative to Google or other internet sources [33]. A chatbot may also provide answers to questions that appear too insignificant or embarrassing to ask health personnel directly [33], potentially reducing the barriers for contacting the health care service [33,42,43].

As there is currently a lack of a standardized methods for evaluating health chatbots, a comparison of chatbots performances may be challenging [25,28]. In general, metrics used to measure chatbot performance depend on which purpose the chatbot is designed to serve; still, most developers aim to keep the conversations short and effective [44]. Keeping this in mind, our results may prove an effective change in user interface in Dina version 2.0, as findings indicate that this version requires fewer conversational steps from the user, evidenced by both fewer questions from users on maneuvering in the chatbot and a decrease in dialogues containing 4-6 questions. This is supported by findings from a previous qualitative study, in which participants stated that they perceived Dina version 2.0 to be effective in providing answers [33]. However, short dialogues could also be an indication of users “giving up” and leaving the conservation; nonetheless, efficiency and the ability to provide a fast answer are important for the intention to use a chatbot [33].

Despite the fact that Dina the chatbot was integrated into the Norwegian digital health platform and made available for all pregnant Norwegian women between the 2 periods of registration, we found that the weekly mean number of dialogues was 36 in the first period and 26.83 in the second period. Although this change was not significant, it could be explained by a possible insufficient promotion of the chatbot among both pregnant women and health care personnel [33]. Previous studies have described several obstacles like organizational, economic, and knowledge barriers when implementing new technology in the health care service [45]. It is our view that the promotion of the chatbot should be a priority going forward to increase the chance of implementation of the chatbot in Norwegian antenatal care.

#### The Chatbot´s Ability To Answer and Need for Further Development

As the chatbot currently does not provide users with the opportunity to express if they are satisfied with the answer, we used the percentage of questions that the chatbot was unable to answer (fallback) as a measure of how well it operates. Our findings showed that a fallback message was given in 11.49% (326/2838) of all questions asked to Dina the chatbot. The goal is to keep the percentage of fallback as low as possible in order to meet user satisfaction [46]. In a previous study on conversational repair in a chatbot developed for customer service, the fallback percentage was reported to be 15% [47]. However, we did not consider “false positive” responses, where the chatbot seemingly provided an answer but not the answer the user sought. This would have provided more insight into the chatbots ability to answer and will be important to consider in future analyses. We categorized questions resulting in a fallback message to discover problem areas that need further development [48] and to satisfy our specific aim of determining what type of questions the chatbot is unable to answer. Our results (Figure 5) suggested an increase in the fallback category “GDM” between the 2 periods of registration. These are questions the chatbot should be able to answer, and calls for further training and development of content regarding information on GDM are warranted. Notably, we found that that number of questions that the chatbot did not understand decreased in the second period, which may be an indication of increased functionality in Dina version 2.0, as the chatbot may provide more options to click on related themes, thereby guiding the user in a more efficient way. Nearly 1 in 4 questions resulting in a fallback message were related to pregnancy in general, and this may be viewed as an expression of an interest from users and possibly serve as an idea for future development. Nonsensical questions represented 16.9% (55/326) of the overall questions resulting in a fallback message from the chatbot, and one could therefore consider the real fallback rate for the chatbot to be lower than 11.5% if one disregarded these questions.

There are several challenges when it comes to integrating this type of new technology into the established health care [39]. Studies outline several reasons as to why users of health technology tools such as chatbots can lose interest, among them being frustrations with the technology and losing the in-person contact with the caregiver [49,50]. A chatbot is not a finished product once it is deployed, and it is important to continuously monitor and add information to improve the chatbot’s ability to function optimally [51]. By highlighting areas in Dina the chatbot that need improvement, we hope this study may serve as a contribution for further improvement and implementation.

### Strength and Limitations

The strengths of this study are its use of the chatbot log data from 2 different periods during the course of continuous development and maintenance of the chatbot [52]. Utilization of user data is a cost-effective approach, providing important insight needed for further development. This study also has some limitations. We were unable to assess if users were women with GDM; however, the chatbot could also be a resource for health personnel or partners or next of kin. As all questions to the chatbot are displayed as dialogues in the chatbot’s log, we were not able to stratify our analysis according to which method the user decide upon (ie, free text, theme buttons, or a mix of the 2). A registration of users’ preferred way of asking questions should be considered in the future development of the chatbot. Furthermore, we could not identify if the user visited the chatbot once or several times. There is currently no way for the users to express whether they received the answer to their question in the dialogue, and obtaining this information would have been a useful addition to the fallback percentage acquired in analyses. Moreover, it would be useful to explore “false positive” responses by the chatbot (the chatbot provides an answer but not the answer the user is seeking). This would have provided more insight into the functionality of the chatbot and will be considered in future studies. The chatbot is currently only available in Norwegian language, limiting the external validity of the knowledge. In addition, not all women may feel comfortable trusting a chatbot on health issues, which might have potentially excluded some women and introduced selection bias into our study.

### Implications

Our findings indicate that users seek information on topics relevant to them at the time, such as blood glucose, diet, and physical activity, and that the most frequently asked questions mirror the cornerstones of GDM treatment. This may indicate that the chatbot is used to quickly access information already provided to users by the health care service, but the chatbot offers a low-threshold way to access that information.

Furthermore, results indicate that Dina version 2.0 guides the user in a more efficient way. However, the low mean number of dialogues per week in the second period (26.83) suggests further efforts should be put into promoting and integrating the chatbot into Norwegian antenatal care. We view our findings as potentially relevant to future development of informational and supportive health chatbots. The authors finds a low-threshold design is an advantage, as this will provide easy access to information the user is also provided through other channels in the health service to further support self-efficacy. As our society quickly becomes more digital, there is a call for the health care service to keep up with the rapid development [36]. We see the need for an informational tool like Dina the chatbot to contribute to increased self-efficacy and coping with GDM, considering the rising prevalence of the condition [2,4,5]. The next step for us with Dina the chatbot would be to continue the ongoing work of further development and to improve promotion to increase its use. We believe that when chatbots are a more integrated part of the health care service, they may serve as a positive contribution to antenatal care.

### Conclusions

The majority of posed questions pertained to blood glucose, diet, the GDM diagnosis, and physical activity, and the chatbot was able to answer about 9 out of 10 of all questions from the users. The most frequent use was during the daytime, Monday through Friday, and the majority of dialogues were short, containing 1-3 questions. However, the mean number of dialogues per week was 36 in the first period and 26.83 in the second period.

## Abbreviations

GDM: gestational diabetes mellitus
